# Serum Peroxiredoxin 4: A Marker of Oxidative Stress Associated with Mortality in Type 2 Diabetes (ZODIAC-28)

**DOI:** 10.1371/journal.pone.0089719

**Published:** 2014-02-25

**Authors:** Esther G. Gerrits, Alaa Alkhalaf, Gijs W. D. Landman, Kornelis J. J. van Hateren, Klaas H. Groenier, Joachim Struck, Janin Schulte, Reinold O. B. Gans, Stephan J. L. Bakker, Nanne Kleefstra, Henk J. G. Bilo

**Affiliations:** 1 Department of Internal Medicine, Maastricht University Medical Center, Maastricht, The Netherlands; 2 Diabetes Centre, Isala Clinics, Zwolle, The Netherlands; 3 Department of Gastroenterology, Isala Clinics, Zwolle, The Netherlands; 4 Langerhans Medical Research Group, Zwolle, The Netherlands; 5 Department of General Practice, University Medical Center Groningen, Groningen, The Netherlands; 6 Thermo Fisher Scientific, BRAHMS Biomarkers, Research Department, Hennigsdorf, Germany; 7 Department of Internal Medicine, University Medical Center Groningen, Groningen, The Netherlands; 8 Department of Internal Medicine, Isala Clinics, Zwolle, The Netherlands; Children's Hospital Boston/Harvard Medical School, United States of America

## Abstract

**Background:**

Oxidative stress plays an underlying pathophysiologic role in the development of diabetes complications. The aim of this study was to investigate peroxiredoxin 4 (Prx4), a proposed novel biomarker of oxidative stress, and its association with and capability as a biomarker in predicting (cardiovascular) mortality in type 2 diabetes mellitus.

**Methods:**

Prx4 was assessed in baseline serum samples of 1161 type 2 diabetes patients. Cox proportional hazard models were used to evaluate the relationschip between Prx4 and (cardiovascular) mortality. Risk prediction capabilities of Prx4 for (cardiovascular) mortality were assessed with Harrell’s C statistic, the integrated discrimination improvement and net reclassification improvement.

**Results:**

Mean age was 67 and the median diabetes duration was 4.0 years. After a median follow-up period of 5.8 years, 327 patients died; 137 cardiovascular deaths. Prx4 was associated with (cardiovascular) mortality. The Cox proportional hazard models added the variables: Prx4 (model 1); age and gender (model 2), and BMI, creatinine, smoking, diabetes duration, systolic blood pressure, cholesterol-HDL ratio, history of macrovascular complications, and albuminuria (model 3). Hazard ratios (HR) (95% CI) for cardiovascular mortality were 1.93 (1.57 – 2.38), 1.75 (1.39 – 2.20), and 1.63 (1.28 – 2.09) for models 1, 2 and 3, respectively. HR for all-cause mortality were 1.73 (1.50 – 1.99), 1.50 (1.29 – 1.75), and 1.44 (1.23 – 1.67) for models 1, 2 and 3, respectively. Addition of Prx4 to the traditional risk factors slightly improved risk prediction of (cardiovascular) mortality.

**Conclusions:**

Prx4 is independently associated with (cardiovascular) mortality in type 2 diabetes patients. After addition of Prx4 to the traditional risk factors, there was a slightly improvement in risk prediction of (cardiovascular) mortality in this patient group.

## Introduction

The importance of identifying patients with diabetes mellitus at high risk for cardiovascular events and mortality has been pointed out extensively in guidelines of the ADA/AHA and ESC/EASD [1], [2]. Risk assessment based on biomarkers such as NT-proBNP, and imaging such as coronary artery calcium imaging and carotid intima-media thickness might be useful markers possibly adding predictive value to traditional risk estimates. Important atherogenic factors in the development of diabetes-related morbidity and mortality are advanced glycation endproducts (AGEs) formed by nonenzymatic glycation of proteins and during oxidative stress [3]. The hyperglycemic state, AGEs and the interaction of AGEs with its receptor RAGE (the so-called AGE-RAGE axis) induce production of reactive oxygen species and is thought to play an important underlying pathophysiologic role in the development of diabetes complications, both microvascular and cardiovascular [4]–[7].

All aerobic organisms have a number of antioxidant proteins as a protection mechanism against oxidative stress. Peroxiredoxin enzymes are thiol-dependent peroxidases and part of a family of proteins present in aerobic organisms, responsible for the degradation of endogenously generated peroxides [8]–[10]. These peroxiredoxin family members are distributed to potential sites of reactive oxygen radicals production: the cytosol, mitochondria, peroxisomes and in plasma [8]. Overexpression or upregulation of peroxiredoxins is associated with higher levels of oxidative stress, suggesting a secondary respons of peroxiredoxins to oxidative stress [8], [11]–[13]. Six isoforms of peroxiredoxins have been described in mammals; peroxiredoxin 4 (Prx4) is the only isoform detectable in serum [14]. Animal models of diabetes mellitus have shown changes of expression or oxidation state of Prx4 in pancreatic islet cells [13], [15]–[17]. No serum levels of Prx4 have been measured before in animal models neither in human with type 2 diabetes mellitus (T2DM). Serum Prx4 has been proposed as a biomarker of oxidative stress in patients with a sepsis [18], [19]. Synovial tissue Prx4 showed an increased expression in patients with rheumatoid arthritis, another inflammatory disease [20]. Recently, Prx4 have been studied in a general Dutch sample, drawn from the general population and included subjects with microalbuminuria [21]. This study showed that elevated serum Prx4 levels were associated with a higher risk of cardiovascular diseases, cardiovascular mortality as well as all-cause mortality and slightly improved risk prediction. The aim of this study was to prospectively investigate whether Prx4 is independently associated with cardiovascular and all-cause mortality and whether it could potentially be a new cardiovascular biomarker in patients with T2DM.

## Materials and Methods

### Study group and design

The included patients in our study are type 2 diabetic patients participating in a shared care project of the Zwolle Outpatient Diabetes project Integrating Available Care (ZODIAC) study. This project started in 1998 in Zwolle, The Netherlands, is still ongoing and has been described in detail previously [22]. In short, the objective of the ZODIAC study was to investigate the effects of a shared-care project for type 2 diabetic patients. Sixty-one general practitioners participated. The present study incorporates two cohorts from the ZODIAC study. The first cohort contained 1143 patients and started in 1998. The second cohort contained 546 patients and started in 2001, leaving a combined cohort of 1689 unique patients included in the current study. Baseline characteristics and mortality rates of the first cohort were similar to these of the second cohort. The ZODIAC study was approved by the medical ethics committee of the Isala Clinics in Zwolle, the Netherlands and all patients gave their written informed consent.

### Data collection

Clinical data were obtained from medical records at the time of inclusion in the ZODIAC study, which consisted of a complete medical history including macrovascular complications, medication use, diabetes duration and smoking history. Patients were considered to have macrovascular complications when they had a history of angina pectoris, myocardial infarction, percutaneous transluminal coronary angioplasty, coronary artery bypass grafting, stroke or transient ischemic attack. Laboratory and physical assessment data, such as glycated hemoglobin (HbA_1c_), non-fasting lipid profile, serum creatinine, albuminuria (albumin-to-creatinine ratio), body mass index (BMI), and blood pressure were collected annually. Blood pressure was measured twice with a Welch Allyn Sphygmomanometer in the supine position after at least five minutes of rest. For each visit the mean blood pressure of two recordings was calculated.

Of the 1689 included patients, 1374 samples were eligible for further analyses to measure Prx4. Complete information on Prx4 and potential confounders in this patient group was available for 1161 patients.

### Serum Peroxiredoxin 4

Prx4 levels were measured in non-fasting serum samples collected at baseline and stored at –80°C until analysis in 2010. Because the performance of one freeze-thaw cycle has no consequences for assessing Prx4 levels, no influence of frozen storage on the assessed levels is to be expected [18]. Prx4 levels were measured with a validated immunoluminometric sandwich assay [18]. In the development of this assay, immunogenic peptides were selected from the N-terminal part of the Prx4 epitope. The immunoassay uses two monoclonal mouse antibodies both directed against amino acids 39 to 51 at this N-terminus of human Prx4. According to the epitope distribution on the Prx4 amino acid sequence, the assay is useable for detection of Prx4 homomultimers and it excludes cross-reactivity with other members of the Prx family.

The functional assay sensitivity (interassay coefficient of variation <20%) is 0.51 arbitrary U/L and the intraassay coefficient of variation is <8%. The assay reports Prx4 concentration as arbitrary units per liter (U/L) and the limit of quantitation was 0.38 arbitrary U/L.

### Clinical endpoints

The clinical endpoints were cardiovascular and all-cause mortality. In 2009, survival status and causes of death were obtained from the local hospital information system and the general practitioners concerning the ZODIAC cohort of 1998. Survival status and causes of death of the ZODIAC cohort of 2001 were obtained in 2005. Causes of death were coded according to the International Classification of Diseases, ninth revision (ICD-9).

### Statistical analyses

SPSS version 16.0 (SAS Insitute, Cary, NC, USA) and STATA version 11 (StataCorp, College Station, Texas USA) were used for statistical analyses. Continuous variables are represented as mean (standard deviation - SD) for normally distributed values and as median (interquartile range - IQR) for non-normally distributed variables. Prx4 levels below 0.38 arbitrary U/L were replaced by an at random generated value between 0 and 0.38.

Cox proportional hazard models were used to investigate the association between Prx4 and (cardiovascular) mortality. The selected variables with possible confounding effects were age, gender, BMI, serum creatinine, smoking, diabetes duration, systolic blood pressure, cholesterol-HDL ratio, history of macrovascular complications, and albuminuria. Four models were chosen: a crude model including only Prx4 (model 1), a model with age and gender as additional confounders (model 2), a fully adjusted model (model 3), and finally a model that contained all the selected confounders except Prx4 (model 4).

Prx4 and serum creatinine were logarithmically transformed because of skewed distribution of the data.

The ph-test was used in combination with inspection of the Schoenfeld residuals to test the assumption of proportional hazards at baseline. Calibration was investigated using the Groennesby and Borgan test, assessing the goodness of fit and determining how well the predicted probabilities agree with the observed risk. When the average predicted risk matches the proportion that actually develops disease within subgroups of a prospective cohort, the model is considered well calibrated [23]. In case of a significant association between Prx4 and (cardiovascular) mortality, the following analyses were performed. Harrell’s C statistic, a rank-based measure, was used to compare how well the different models predict mortality [24]. The higher the value the better the model predicts mortality. Furthermore, the integrated discrimination improvement (IDI) and the net reclassification improvement (NRI) using 10%, 20%, and 30% cut-off values, were calculated [25]. Both, the IDI and the NRI are designed to evaluate the improvement in prediction by novel markers. They can be interpreted as the difference between model-based probabilities for events and non events for models with and without Prx4. The IDI is a global measure of correct reclassification regarding all possible cut-off values, while the NRI gives the reclassification improvement for a number of cut-off values. The 95% confidence intervals for Harrell’s C, IDI, and NRI are given in the results.

## Results

Baseline characteristics of the 1161 included patients are presented in strata according to Prx4 levels (table 1). 137 Patients had a Prx4 level below 0.38 arbitrary U/L. Median (interquartile range, IQR) serum level of Prx4 was 0.79 (0.53 – 1.25) arbitrary U/L. Patients with Prx4 above the median were older, had a higher BMI, lower eGFR, lower HDL cholesterol levels, higher HbA_1c_ levels, higher prevalence of albuminuria and less frequently received lipid-lowering drugs, although the percentage of smokers was lower. After a median (IQR) follow-up of 5.8 (3.1– 10.1) years, 327 (28%) patients had died, of which 137 (42%) were attributable to cardiovascular disease.

**Table 1:** Baseline characteristics.

Baseline characteristic	Total n = 1161	Group 1 Prx4 < median*	Group 2 Prx4 > median	p-value
**Age (years)**	67 (12)	65 (11)	68 (11)	<0.001^1^
**Gender male (%)**	522 (45)	276 (48)	246 (42)	0.09^3^
**Diabetes duration (years)**	4.0 [2.0 – 9.0]	4.0 [2.0 – 9.0]	4.0 [2.0 – 9.0]	0.43^2^
**eGFR (Cockcroft-Gault) (ml/min) [n = 1022]**	72 [57 – 92]	73 [58 – 94]	70 [55 – 90]	0.008^2^
**BMI (kg/m^2^)**	28.7 [25.8 – 32.0]	28.1 [25.4 – 31.3]	29.2 [26.0 – 32.6]	<0.001^2^
**Smoking (%)**	220 (19)	125 (22)	95 (16)	0.03^3^
**Systolic blood pressure (mmHg)**	152 (24)	151 (24)	153 (24)	0.37^1^
**HbA1c (%)**	7.0 [6.2 – 8.1]	6.8 [6.2 – 7.9]	7.1 [6.3 – 8.2]	0.01^2^
**Albuminuria (%)**	455 (39)	182 (31)	273 (47)	<0.001^3^
**Total cholesterol (mmol/l)**	5.4 [4.8 – 6.2]	5.5 [4.8 – 6.2]	5.4 [4.7 – 6.2]	0.25^2^
**HDL – cholesterol (mmol/l)**	1.2 [1.0 – 1.4]	1.2 [1.0 – 1.4]	1.1 [0.9 – 1.3]	0.003^2^
**Cholesterol – HDL ratio**	4.7 [3.8 – 5.8]	4.7 [3.8 – 5.6]	4.8 [3.9 – 6.0]	0.02^2^
**Macrovascular complications (%)**	413 (36)	195 (34)	218 (38)	0.16^3^
**Receiving lipid-lowering drugs (statins) (%)**	187 (16)	110 (19)	77 (13)	0.01^3^
**Receiving trombocyte aggregation inhibitor (%)**	185 (16)	92 (16)	93 (16)	0.94^3^
**Receiving ACE-I/ARB (%)**	313 (27)	160 (28)	153 (26)	0.69^3^

Abbreviations: eGFR, estimated glomerular filtration rate; BMI, body mass index; HbA1c, glycated haemoglobin; HDL, high-density lipoprotein; ACE-I, angiotensin converting enzyme inhibitor; ARB, angiotensin receptor blocker.

*) Median Prx4 (U/L): 0.79 [0.53 – 1.25].

Data are means (SD), medians [interquartile range], or n (%). P-values show (non)significance of group 2 compared to group 1. 1) Student’s t-test. 2) Mann-Whitney U test. 3) Fisher’s Exact test.

The survivors had lower median (IQR) baseline levels of Prx4 compared to the non-survivors [0.71 (0.48 – 1.05) arbitrary U/L] versus [1.05 (0.65 – 1.59) arbitrary U/L]; p<0.001.

Inspection of the Schoenfeld residuals and Stata’s ph-test showed no violations of the assumption of proportional hazards. The goodness of fit analyses indicated that the models for cardiovascular mortality as well as for all-cause mortality were well calibrated.

Increased levels of Prx4 were associated with higher rates of cardiovascular and all-cause mortality (Table 2). These associations persisted after adjustment for confounders in models 2 and 3.

**Table 2:** Hazard ratios for cardiovascular and all-cause mortality of the logarithmically transformed Prx4.

	Model 1	Model 2	Model 3	Model 4
**Cardiovascular mortality**				
Hazard ratio [95% CI]	1.93 [1.57 – 2.38]	1.75 [1.39 – 2.20]	1.63 [1.28 – 2.09]	n.a.
Harrell’s C [95% CI]	0.65 [0.61 – 0.70]	0.77 [0.73 – 0.81]	0.82 [0.78 – 0.85]	0.81 [0.77 – 0.84]
IDI % [95% CI] p-value	n.a.	1.97 [1.03 – 2.91] 0.00004	0.97 [0.16 – 1.77] 0.018	n.a.
NRI % [95% CI]	n.a.	11.78 [2.35 – 21.21]	6.48 [0.00 – 13.33]	n.a.
**All-cause mortality**				
Hazard ratio [95% CI]	1.73 [1.50 – 1.99]	1.50 [1.29 – 1.75]	1.44 [1.23 – 1.67]	n.a.
Harrell’s C [95% CI]	0.64 [0.61 – 0.67]	0.79 [0.76 – 0.81]	0.81 [0.78 – 0.83]	0.80 [0.77 – 0.82]
IDI % [95% CI] p-value	n.a.	2.38 [1.41 – 3.34] < 0.00001	1.63 [0.82 – 2.44] 0.00009	n.a.
NRI % [95% CI]	n.a.	6.82 [2.42 – 11.23]	8.03 [3.81 – 12.24]	n.a.

Comparison of predictive capability of models for mortality risk prediction as determined by the Harrell’s C statistic, the IDI, and NRI.

Abbreviations: Prx4, peroxiredoxin 4; HR, hazard ratio; CI, confidence interval; IDI, integrated discrimination improvement; NRI, net reclassification improvement; NA, not applicable.

Cox regression models: Model 1: crude model with Prx4; Model 2: adjusted for age and gender; Model 3: adjusted for age, gender, smoking (dichotomous), body mass index, systolic blood pressure, duration of diabetes, serum creatinine level, cholesterol-HDL ratio, macrovascular complications (dichotomous), albuminuria (dichotomous); Model 4: all selected confounders without Prx4.

The Harrel’s C values, as presented in Table 2, show that with increasing numbers of confounders, the better the model predicted cardiovascular mortality and all-cause mortality. However, no differences in the C values were observed between models 3 (the fully adjusted model) and model 4 (the fully adjusted model without Prx4).

Furthermore, the IDI and NRI were positive in models 2 and 3 for both cardiovascular and all-cause mortality.

Concerning cardiovascular mortality, changes in classification for Prx4 with age and gender are shown in table 3 and with all known risk factors in table 4. For example: without adjusting for Prx4, 25 patients had been classified in the cardiovascular mortality risk group of < 10%. However, when Prx4 was added to this risk model, 6 patients were reclassified into the cardiovascular mortality risk group of 10 – 20%. Furthermore, out of a total number of 577 patients in the survivor group: 41, 2 and 1 patient(s) were reclassified into another category after adding Prx4 to the model.

**Table 3:** Changes in classification for Prx4 with age and gender (cardiovascular mortality).

	Age, gender and Prx4				
Age and gender	< 10%	10 – 20%	20 – 30%	> = 30%	Total
**deceased**					
< 10%	19	6			25
10 – 20%	3	49	13	1	66
20 – 30%		8	16	7	31
> = 30%			2	13	15
Total	22	63	31	21	137
**survivals**					
< 10%	533	41	2	1	577
10 – 20%	64	192	41	1	298
20 – 30%	2	27	62	23	114
> = 30%	2	1	8	24	35
Total	601	261	113	49	1024
NRI % [95% CI]	11.78 [2.35 – 21.21]				

**Table 4:** Changes in classification for Prx4 with all known risk factors (cardiovascular mortality).

	All known risk factors and Prx4				
All risk factors	< 10%	10 – 20%	20 – 30%	> = 30%	Total
**deceased**					
< 10%	21	3			24
10 – 20%	1	29	8		38
20 – 30%		3	20	2	25
> = 30%			4	46	50
Total	22	35	32	48	137
**survivals**					
< 10%	671	22			693
10 – 20%	43	127	14	1	185
20 – 30%	2	10	49	12	73
> = 30%		1	12	60	73
Total	716	160	75	73	1024
NRI % [95% CI]	6.48 [0.00 – 13.33]				

Table 5 and 6 show the changes in classification for Prx4 with age and gender respectively with all known risk factors concerning all-cause mortality.

**Table 5:** Changes in classification for Prx4 with age and gender (all-cause mortality).

	Age, gender and Prx4				
Age and gender	< 10%	10 – 20%	20 – 30%	> = 30%	Total
**deceased**					
< 10%	14	2			16
10 – 20%	2	18	4		24
20 – 30%		4	14	8	26
> = 30%	1	3	6	251	261
Total	17	27	24	259	327
**survivals**					
< 10%	295	12	2		309
10 – 20%	38	118	23	3	182
20 – 30%	7	26	64	16	113
> = 30%	1	10	27	192	230
Total	341	166	116	211	834
NRI% [95% CI]	6.82[2.42 – 11.23]				

**Table 6:** Changes in classification for Prx4 with all known risk factors (all-cause mortality).

	All known risk factors and Prx4				
All risk factors	< 10%	10 – 20%	20 – 30%	> = 30%	Total
**deceased**					
< 10%	19	3			22
10 – 20%	1	8	7		16
20 – 30%	1	4	13	10	28
> = 30%	1	1	4	256	261
Total	21	16	24	266	327
**survivals**					
< 10%	367	17	1	1	386
10 – 20%	34	109	18		161
20 – 30%	1	21	59	11	92
> = 30%		8	18	169	195
Total	402	155	96	181	834
NRI% [95% CI]	8.03 [3.81 – 12.24]				

## Discussion

Our study provides the first evidence that the free serum antioxidant Prx4 is independently associated with cardiovascular and all-cause mortality in patients with T2DM after long-term follow up. Prx4 could play a role in the prediction of (cardiovascular) mortality in T2DM, although there was no relevant added beneficial effect of Prx4 compared to a fully adjusted model. Adding Prx4 to age and gender (model 2) had a comparable predictive value as adding Prx4 to the combined traditional cardiovascular risk factors (model 3).

Two recent studies showed that Prx4 has predictive capabilities [21], [26]. One was established for 30-days mortality in patients with nonspecific complaints presenting at the emergency department and the other study demonstrated the association between Prx4 and cardiovascular morbidity and mortality and all-cause mortality in a large general population with a median follow up time of 10.5 years [21], [26]. No previous studies were performed in patients with DM.

When compared to healthy controls, patients with inflammatory conditions like sepsis had higher levels of circulating Prx4 [18], [19]. Non-surviving patients with a sepsis have higher Prx4 levels compared to the surviving patients and Prx4 is correlated with markers of infection and inflammation, like procalcitonin, C-reactive protein (CRP) and interleukin 6 [19], [20]. Additional information about correlations with antioxidant markers and with oxidative damage markers would be useful to establish the use of Prx4 as a novel biomarker of oxidative stress.

In response to free oxygen radical production in T2DM, secretion of thiol-dependent peroxidases will increase to participate in the removal of these reactive oxygen species. It is hypothesized that the intracellular removal of hydrogen peroxides by Prx4 and secretion of the enzyme is proportionally upregulated in response to the surrounding oxidative stress [10], [14]. Oxidative stress also causes endothelial damage, which possibly could result in additional endothelial tissue leakage of Prx4, contributing to even higher levels of serum Prx4. It still has to be investigated if Prx4 is actively removing hydrogen peroxides in the circulation. Perhaps, Prx4, being part of the antioxidant defense system, can rather be considered as a marker of endothelial cell damage and therefore would indirectly be linked to oxidative stress.

Our study had a few limitations. Patients with higher levels of Prx4 in our study revealed several characteristics that may have influenced Prx4 or oxidative stress in general. These include older age, higher prevalence of albuminuria, higher BMI, lower levels of HDL and less use of lipid lowering drugs like statins. However, even after adjustment for most of these confounders, the associations between Prx4 and mortality remained significant.

Furthermore, our analyses were performed in only 1161 out of the initially 1689 included patients, so selection bias may have occurred. Secondly, because we only adjusted for a single baseline Prx4 value, we were not able to adjust for potential variability in Prx4 concentrations. Whether sequential measurements of Prx4 will result in a better prediction of mortality in patients with diabetes remains to be investigated. Mortality data ranging up to 2009 for the whole population would have been of additional value. We also acknowledge the lack of data on CRP or IL-6 as a limitation. The addition of CRP or IL-6 could have provided insight into the pathophysiology of and the interface between inflammation and oxidative stress. Prx4 might behave like an acute phase reactant, but also as a biomarker, regardless of the causal pathway.

Finally, care must be taken in interpreting the values of the IDI and NRI, because these measures are dependent on the prevalence of the number of events and are not developed in the context of censored data. Besides this, there is no consensus regarding the interpretation of the magnitude of both measures.

Strengths of our study were the number and completeness of confounders, its prospective design and a long follow up period. It is also the first study of Prx4 in a large group of patients, including over 1000 T2DM patients.

To conclude, Prx4 is a circulating antioxidant and is independently associated with increased risk of cardiovascular and all-cause mortality in T2DM. Future studies are needed to answer the question whether Prx4, as a novel biomarker of oxidative stress, may be a new valuable cardiovascular predictor useful for risk stratification in T2DM.

## References

[1] Buse JB, Ginsberg HN, Bakris GL, Clark NG, Costa F, et al (2007) Primary prevention of cardiovascular diseases in people with diabetes mellitus: a scientific statement from the American Heart Association and the American Diabetes Association. Diabetes Care 30: 162–172. 17192355 10.2337/dc07-9917

[2] Rydén L, Grant PJ, Anker SD, Berne C, Cosentino F, et al (2013) ESC guidelines on diabetes, pre-diabetes, and cardiovascular diseases developed in collaboration with the EASD: the task force on diabetes, pre-diabetes, and cardiovascular diseases of the European Society of Cardiology (ESC) and developed in collaboration with the European Association for the Study of Diabetes (EASD). Eur Heart J 34: 3035–87. 23996285 10.1093/eurheartj/eht108

[3] Brownlee M (2005) The pathobiology of diabetic complications: a unifying mechanism. Diabetes 54: 1615–1625. 15919781 10.2337/diabetes.54.6.1615

[4] Rains JL, Jain SK (2011) Oxidative stress, insulin signaling, and diabetes. Free Radic Biol Med 50: 567–575. 21163346 10.1016/j.freeradbiomed.2010.12.006 PMC3557825

[5] Yan SF, Ramasamy R, Schmidt AM (2010) The RAGE axis: a fundamental mechanism signaling danger to the vulnerable vasculature. Circ Res 106: 842–53. 20299674 10.1161/CIRCRESAHA.109.212217 PMC2862596

[6] Rolo AP, Palmeira CM (2006) Diabetes and Mitochondrial function: role of hyperglycemia and oxidative stress. Toxicol Appl Pharmacol 212: 167–178. 16490224 10.1016/j.taap.2006.01.003

[7] Giacco F, Brownlee M (2010) Oxidative stress and diabetic complications. Circ Res 107: 1058–1070. 21030723 10.1161/CIRCRESAHA.110.223545 PMC2996922

[8] Wood ZA, Schröder E, Harris JR, Poole LB (2003) Structure, mechanism and regulation of peroxiredoxins. Trends Biochem Sci 28: 32–40. 12517450 10.1016/s0968-0004(02)00003-8

[9] Valko M, Leibfritz D, Moncol J, Cronin M, Mazur M, et al (2007) Free radicals and antioxidants in normal physiological functions and human disease. Int J Biochem Cell Biol 39: 44–84. 16978905 10.1016/j.biocel.2006.07.001

[10] Tavender TJ, Bulleid NJ (2010) Peroxiredoxin IV protects cells from oxidative stress by removing H_2_O_2_ produced during disulphide formation. J Cell Science 123: 2672–2679. 20627953 10.1242/jcs.067843 PMC2908052

[11] Fujii J, Ikeda Y (2002) Advances in our understanding of peroxiredoxin, a multifunctional, mammalian redox protein. Redox Rep 7: 123–130. 12189041 10.1179/135100002125000352

[12] Rabilloud T, Heller M, Gasnier F, Luche S, Rey C, et al (2002) Proteomics analysis of cellular response to oxidative stress: evidence for in vivo over-oxidation of peroxiredoxins at their active site. J Biol Chem 277: 19396–19401. 11904290 10.1074/jbc.M106585200

[13] Ding Y, Yamada S, Wang KY, Shimajiri S, Guo X, et al (2010) Overexpression of peroxiredoxin 4 protects against high-dose streptozotocin-induced diabetes by suppressing oxidative stress and cytokines in transgenic mice. Antioxid Redox Signal 13: 1477–1490. 20446767 10.1089/ars.2010.3137

[14] Okado-Matsumoto A, Matsumoto A, Fujii J, Taniguchi N (2000) Peroxiredoxin IV is a secretable protein with heparin-binding properties under reduced conditions. J Biochem 127: 493–501. 10731722 10.1093/oxfordjournals.jbchem.a022632

[15] Xie X, Li S, Liu S, Lu Y, Shen P, Ji J (2008) Proteomic analysis of mouse islets after multiple low-dose streptozotocin injection. Biochim Biophys Acta 1784: 276–284. 18082151 10.1016/j.bbapap.2007.11.008

[16] Dreja T, Jovanovic Z, Rasche A, Kluge R, Herwig R, et al (2010) Diet-induced gene expression of isolated pancreatic islets from a polygenic mouse model of the metabolic syndrome. Diabetologia 53: 309–320. 19902174 10.1007/s00125-009-1576-4 PMC2797618

[17] Jiang YL, Ning Y, Ma XL, Liu YY, Wang Y, et al (2011) Alteration of the proteome profile of the pancreas in diabetic rats induced by streptozotocin. Int J Mol Med 28: 153–160. 21567075 10.3892/ijmm.2011.696

[18] Schulte J, Struck J, Bergmann A, Köhrle J (2010) Immunoluminometric assay for quantification of peroxiredoxin 4 in human serum. Clin Chim Acta 411: 1258–1263. 20493180 10.1016/j.cca.2010.05.016

[19] Schulte J, Struck J, Köhrle J, Müller B (2011) Circulating levels of peroxiredoxin 4 as a novel biomarker of oxidative stress in patients with sepsis. Shock 35: 460– 465. 10.1097/SHK.0b013e3182115f40 21283059

[20] Chang X, Cui Y, Zong M, Zhao Y, Yan X, et al (2009) Identification of proteins with increased expression in rheumatoid arthritis synovial tissues. J Rheumatol 36: 872–880. 19369474 10.3899/jrheum.080939

[21] Abbasi A, Corpeleijn E, Postmus D, Gansevoort RT, de Jong PE, et al (2012) Peroxiredoxin 4, a novel circulating biomarker for oxidative stress and the risk of incident cardiovascular disease and all-cause mortality. J Am Heart Assoc 1: e002956. 23316297 10.1161/JAHA.112.002956 PMC3541606

[22] Ubink-Veltmaat LJ, Bilo HJ, Groenier KH, Houweling ST, Rischen RO, et al (2003) Prevalence, incidence and mortality of type 2 diabetes mellitus revisited. A prospective population-based study in The Netherlands (ZODIAC-1). Eur J of Epidemiol 18: 793–800. 12974556 10.1023/a:1025369623365

[23] May S, Hosmer DW (1998) A simplified method of calculation an overall goodness of-fit test for the Cox proportional hazards model. Lifetime Data Anal 4: 109–120. 9658770 10.1023/a:1009612305785

[24] Harrell FE Jr, Lee KL, Mark DB (1996) Multivariable prognostic models: issues in developing models, evaluating assumptions and adequacy, and measuring and reducing errors. Stat Med 15: 361–387. 8668867 10.1002/(SICI)1097-0258(19960229)15:4<361::AID-SIM168>3.0.CO;2-4

[25] Pencine MJ, D’Agostino RB, Vasan RS (2010) Statistical methods for assessment of added usefulness of new biomarkers. Clin Chem Lab Med 48: 1703–1711. 20716010 10.1515/CCLM.2010.340 PMC3155999

[26] Nickel CH, Ruedinger J, Misch F, Blume K, Maile S, et al (2011) Copeptin and Peroxiredoxin-4 independently predict mortality in patients with nonspecific complaints presenting to the emergency department. Acad Emerg Med 18: 851–859. 21843221 10.1111/j.1553-2712.2011.01126.x

